# Supplementary material for: Population pharmacokinetics of DNDI-6148 in healthy adults
Source: PLoS Negl Trop Dis. 2026 Apr 20;20(4):e0014220. doi: 10.1371/journal.pntd.0014220 (PMC13138750; doi:10.1371/journal.pntd.0014220)
Supplement: S1 Fig — (DOCX) [file pntd.0014220.s001.docx]

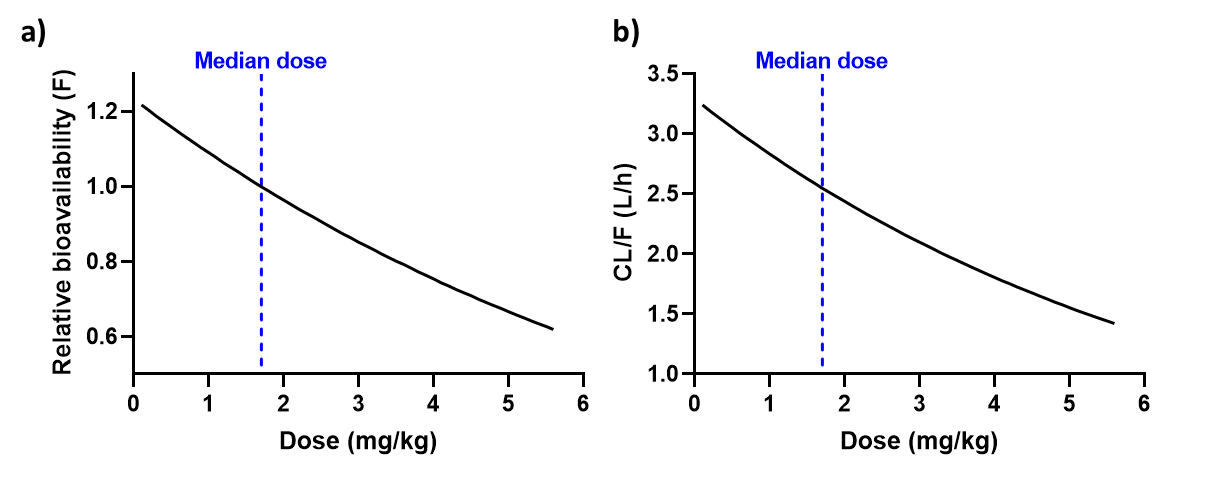


**S1 Fig.** **Covariate effects of dose on DNDI-6148 pharmacokinetic parameters**

Panels show the estimated impact of dose (mg/kg) on a) relative bioavailability (F) and b) apparent clearance (CL/F) in the single-ascending-dose study. Relationships were described using exponential functions, with estimated exponents of –0.123 for F and –0.15 for CL/F. The vertical dashed line marks the median dose in the current study (1.7 mg/kg).
