# Supplementary material for: Population pharmacokinetics of DNDI-6148 in healthy adults
Source: PLoS Negl Trop Dis. 2026 Apr 20;20(4):e0014220. doi: 10.1371/journal.pntd.0014220 (PMC13138750; doi:10.1371/journal.pntd.0014220)
Supplement: S2 Fig — (DOCX) [file pntd.0014220.s002.docx]

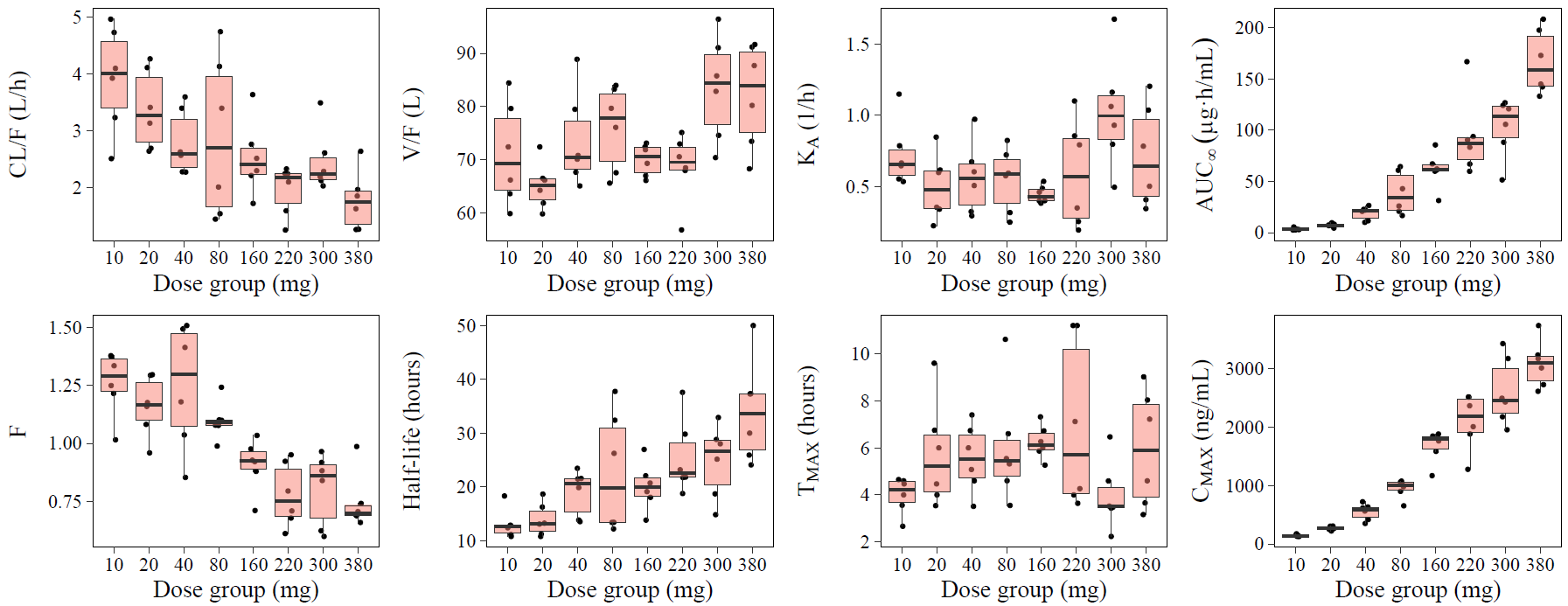


**S2 Fig.** **Distribution of individual primary and secondary plasma PK parameters of DNDI-6148 by dose level.** The midline in the boxes indicates the median; the box shows the interquartile range (IQR); whiskers extend to 1.5 × IQR.
