# Supplementary material for: Population pharmacokinetics of DNDI-6148 in healthy adults
Source: PLoS Negl Trop Dis. 2026 Apr 20;20(4):e0014220. doi: 10.1371/journal.pntd.0014220 (PMC13138750; doi:10.1371/journal.pntd.0014220)
Supplement: S1 Code — (DOCX) [file pntd.0014220.s003.docx]

**S1 Code. NONMEM code of the final population PK model**

;;-------------------------------------------------------------------------------------------------------------;;

$SUBROUTINE ADVAN5 TRANS1

;;-------------------------------------------------------------------------------------------------------------;;

$MODEL

COMP=(1) ; Absorption compartment

COMP=(2) ; Central compartment

;;-------------------------------------------------------------------------------------------------------------;;

$PK

; Dose effect

COV = EXP(THETA(5)*(DOSE_P_KG - 1.7))

COV2 = EXP(THETA(6)*(DOSE_P_KG - 1.7))

;;-------------------------------------------------------------------------------------------------------------;;

; Disposition parameters

TVCL = THETA(1) *((WT/70)**0.75) *COV2 ; Elimination clearance (parent)

CL = TVCL_P *EXP(ETA(1))

TVV2 = THETA(2) *((WT/70)**1.00) ; Central volume (parent)

V2 = TVV2 *EXP(ETA(2))

TVKA = THETA(3) ; Absorption rate

KA = TVKA *EXP(ETA(3))

TVF1 = THETA(4) *COV ; Relative bioavailability

F1 = TVF1 *EXP(ETA(4))

K12 = KA

K20 = CL/V2

S2 = V2

;;-------------------------------------------------------------------------------------------------------------;;

$ERROR

CP = A(2)/S2

IPRED = CP

IF(IPRED.GT.0) IPRED = LOG(IPRED)

Y = IPRED + EPS(1)

W = SQRT(SIGMA(1,1))

IRES = DV-IPRED

IWRES = IRES/W

IF(AMT.GT.0) DTIM=TIME

TAD=TIME-DTIM

;;-------------------------------------------------------------------------------------------------------------;;

$THETA (0, 2.55) ; 1 CL/F (L/h)

$THETA (0, 69.9) ; 2 V/F (L)

$THETA (0, 0.576) ; 3 K_A_ (h^-1^)

$THETA (1) FIX ; 4 F

$THETA (-0.123) ; 5 Dose effect on F

$THETA (-0.15) ; 6 Dose effect on CL/F

;;-------------------------------------------------------------------------------------------------------------;;

$OMEGA 0.0905 ; 1 IIV_CL/F

$OMEGA 0 FIX ; 2 IIV_V/F

$OMEGA 0.274 ; 3 IIV_K_A_

$OMEGA 0.0284 ; 4 IIV_F

;;-------------------------------------------------------------------------------------------------------------;;

$SIGMA 0.0361 ; Additive residual error

;;-------------------------------------------------------------------------------------------------------------;;

$ESTIMATION

MAXEVAL=9999 PRINT=1 METHOD=1 INTER MCETA=50
