# Supplementary material for: Population pharmacokinetics of DNDI-6148 in healthy adults
Source: PLoS Negl Trop Dis. 2026 Apr 20;20(4):e0014220. doi: 10.1371/journal.pntd.0014220 (PMC13138750; doi:10.1371/journal.pntd.0014220)
Supplement: Attachment — Submitted filename: Response_to_Reviewers.pdf [file pntd.0014220.s005.pdf]

Response to Reviewers

Responses to reviewer comments are highlighted in blue.

Reviewer #1

Methods, Results, Conclusions, Editorial & Data Presentation Modifications?

see comments

Summary and General Comments

Assmus and colleagues have fitted a population PK model to previously published healthy volunteer data on the PK of DNDI-6148. Finding dose proportional AUC but decreasing Cmax they fit a model with dose decreasing bioavailability, offset with decreasing CL/F. The paper is clearly written and easy to follow and the model seems to describe the data well with precisely-estimated parameters. Authors should consider the following:

Comments

Title could be shortened to: Population Pharmacokinetics of DNDI-6148 in Healthy Adults.

Thank you for the suggestion.

We have shortened the title of the manuscript to 'Population Pharmacokinetics of DNDI-6148 in Healthy Adults', as recommended.

Line 120: please provide assay lower limit of quantification and precision.

The lower limit of quantification (LLOQ) of the assay was 1 ng/mL, and is stated in the Methods section (*Pharmacokinetic sample collection and drug quantification*).

We have now added assay precision information: "Between-run and within-run assay precision were ≤10.10% and ≤4.63% CV (coefficient of variation), respectively."

Line 141: please justify fixing %CV values <10% to zero with a reference.

We have removed the general statement regarding fixing inter-individual variability values below a predefined threshold from the Methods section. Instead, we now explicitly clarify the rationale in the Results.

Sentence added to Results (*Population pharmacokinetic model*): "Inter-individual variability on apparent volume of distribution was estimated to be close to zero and was therefore fixed to zero in the final model."

Line 148: please write out the exact dose function. Others have used mg/m2 or allometric 0.75 since this is more likely to scale with gastric surface area and extrapolate to special populations e.g. children. Please also justify scaling with linear body weight.

We thank the reviewer for this comment and clarify the implementation of body weight–based allometric scaling and dose covariate effects in the model.

We do not suggest that dosing should scale linearly with bodyweight. In fact, allometric scaling of body weight was incorporated a priori on all clearance and volume parameters to account for the nonlinear relationship between weight and clearance. This ensured that body-size effects on drug disposition were appropriately handled prior to the evaluation of different covariates.

All covariates were evaluated using a common stepwise approach. Dose (mg/kg) was a statistically significant covariate on relative bioavailability, implemented as an exponential function. A decrease in the extent of absorption of drugs with low solubility is commonly observed with increasing doses. After addition of this covariate effect, dose (mg/kg) was also selected as a statistically significant covariate on CL/F. The apparent decrease in clearance and prolongation of half-life at higher doses may reflect saturation of hepatic processes, although other contributing factors cannot be excluded (see Discussion section).

For completeness, dose was also evaluated as total dose (mg). However, the mg/kg formulation provided a better statistical fit than total dose (lower OFV) and more consistently captured the nonlinear relationship between dose and exposure. Moreover, expressing dose on an mg/kg basis facilitates comparison of dose effects across body sizes and provides a framework for extrapolation to paediatric populations. Such extrapolation should nevertheless be interpreted with caution, as the present analysis was based on a Phase 1 study in healthy adult volunteers.

The specific dose–covariate relationships (expressed as mg/kg) are presented in the Results section (Eq. 1 and Eq. 2), and the complete NONMEM implementation is provided in the Supplementary Information (Code S1).

Overall, the combined handling of nonlinear body-size effects on disposition and dose-dependent effects on exposure provides a framework for the exploration of dosing regimens in subsequent analyses.

Line 152: Are authors saying they tested each covariate three times as a linear, exponential and power? What is the justification for this e.g. the power and exponential can both approximate linear and each other.

We thank the reviewer for this comment and agree that further clarification was needed. We have refined the Methods section to explicitly state that linear, exponential, and power relationships were evaluated in parallel for all covariates.

We have also expanded the Results section to clarify that, for the retained dose effects on F and CL/F, power functions yielded larger reductions in OFV but resulted in steep relationships at low doses. Exponential functions were therefore selected for the final model due to their more conservative behaviour at low doses and improved suitability for translational applications. This rationale is also briefly summarized in the Discussion.

Manuscript changes:

- Sentence refined in Methods (*Covariate selection*): "For all covariates, linear, exponential, and power relationships were evaluated in parallel, using the scm functionality in PsN."
- Text added to Results (*Population pharmacokinetic model*): "In addition, dose (in mg/kg) was identified as a significant covariate on relative bioavailability (F) and subsequently on CL/F. A dose effect on F alone was insufficient to capture the observed increase in terminal elimination half-life at higher doses and approximately dose-proportional AUC_{∞}, necessitating the inclusion of an additional dose effect on CL/F.

  For the retained dose effects on F and CL/F, power functions resulted in larger reductions in OFV but produced steep relationships at low doses. Exponential functions centered on the median dose ($Dose_{median}$ = 1.7 mg/kg) were therefore selected for the final model as a more conservative approach with improved suitability for translational applications."
- Sentence refined in Discussion (*Pharmacokinetic properties of DNDI-6148*): "Dose effects on both CL/F and F were described using exponential functions centered on the median dose, selected as a conservative and physiologically reasonable approximation after evaluation of linear, power, and E_{MAX} relationships."

Line 209: Unclear why two equations required given only oral data used? How are beta_dose_F and beta_dose_CL structurally identifiable?

We thank the reviewer for this important question. Although only oral data were available, two dose-dependent relationships were required to adequately describe the distinct features of the data:

A dose effect (mg/kg) on F was needed to capture the less-than-dose-proportional increase in C_{MAX}, consistent with solubility-limited absorption. However, a dose effect on F alone was insufficient to describe the observed increase in terminal elimination half-life at higher doses and the approximately dose-proportional AUC_{∞}. Inclusion of an additional dose effect on CL/F was therefore necessary to offset the reduced bioavailability and reproduce overall exposure and the terminal slope. Both dose-effect parameters were estimated with acceptable precision (RSEs <15%). The apparent decline in clearance and increase in half-life at higher doses may reflect saturation of hepatic processes, but other factors could contribute (see Discussion section).

Models including only a dose effect on apparent volume of distribution (V/F) provided comparable statistical fits but were considered less physiologically plausible, given the absence of evidence for concentration-dependent tissue binding in preclinical studies.

We acknowledge that the analysis is based on a single-dose oral study and that intravenous data would be required to fully disentangle absorption-related from elimination-related processes.

Manuscript changes:

- Sentence added to Results (*Population pharmacokinetic model*): "A dose effect on F alone was insufficient to capture the observed increase in terminal elimination half-life at higher doses and approximately dose-proportional AUC_{∞}, necessitating the inclusion of an additional dose effect on CL/F."
- Sentence added to Discussion (*Pharmacokinetic properties of DNDI-6148*): "As DNDI-6148 was administered as an oral suspension, it cannot be excluded that dose-dependent processes during the absorption phase contributed to the apparent decrease in clearance at higher doses."
- Sentence added to Discussion (*Strengths and limitations*): "The present analysis is based on oral administration only. While the population PK model adequately described the observed data, intravenous administration would be required to fully disentangle absorption-related from elimination-related processes."

Fig 2: GOF plots require a smooth and CWRES dashed lines at -2 and +2

Thank you for the suggestion. We have added LOESS smoothing lines to the observed versus predicted goodness-of-fit plots and included dashed reference lines at CWRES = −2 and +2 in the CWRES plots.

Table 2: put the equations for the two beta terms into the footnote to aid interpretation of parameters.

Thank you for the suggestion. We have added the equations describing the dose effects on relative bioavailability (F) and apparent clearance (CL/F) to the footnote of Table 2 to aid interpretation of these parameters. To ensure consistency with population pharmacokinetic nomenclature and the model code provided in the Supplementary Information, we have replaced β notation with θ notation in Table 2 and throughout the manuscript.

A further figure showing how typical F and CL/F change with dose i.e. drawing out the two functions, would help readers interpret the way authors have chosen to handle the nonlinearity

Thank you for the suggestion. A figure showing the dose-dependent relationships for F and CL/F is already provided in the Supplementary Information (Figure S1).

We have now made this more explicit in the Results section by adding the following sentence: "To aid interpretation of the implemented dose effects, **Figure S1** illustrates the exponential relationships of F and CL/F as functions of dose used in the final model."

Reviewer #2

Methods

The study design and data analysis approaches are well described.

Results

The applied data analyses are sound and limitations appropriately discussed. Data is well presented in Figures and Tables.

Conclusions

All data is appropriately discussed. The article provides a relevant resource for further DNDI-6148 development and puts the data into context for non-expert readers.

Editorial and Data Presentation Modifications?

(No Response)

Summary and General Comments

Assmus et al. carried out a population pharmacokinetics study for the benzoxaborole DNDI-6148, currently in the DNDi development pipeline for treatment of visceral leishmaniasis. Additionally, the compound was suggested as a candidate drug for cutaneous leishmaniasis (PMID: 30922847) and Chagas disease (PMID: 34711050). The study builds on a FIH Phase 1 study in which DNDI-6148 was shown to be safe and well tolerated after a single oral dose.

The outcome of the population pharmacokinetics study with 48 healthy participants revealed non-linear dose–exposure relationship and will be a valuable resource for clinical trials.

Thank you for your positive and encouraging review.
