# Supplementary material for: Population pharmacokinetics of DNDI-6148 in healthy adults
Source: PLoS Negl Trop Dis. 2026 Apr 20;20(4):e0014220. doi: 10.1371/journal.pntd.0014220 (PMC13138750; doi:10.1371/journal.pntd.0014220)

Response to Reviewer #1

Responses to reviewer comments are highlighted in blue.

Methods

-Are the objectives of the study clearly articulated with a clear testable hypothesis stated?
-Is the study design appropriate to address the stated objectives?
-Is the population clearly described and appropriate for the hypothesis being tested?
-Is the sample size sufficient to ensure adequate power to address the hypothesis being tested?
-Were correct statistical analysis used to support conclusions?
-Are there concerns about ethical or regulatory requirements being met?

Reviewer #1: yes

Results

-Does the analysis presented match the analysis plan?
-Are the results clearly and completely presented?
-Are the figures (Tables, Images) of sufficient quality for clarity?

Reviewer #1: yes

Conclusions

-Are the conclusions supported by the data presented?
-Are the limitations of analysis clearly described?
-Do the authors discuss how these data can be helpful to advance our understanding of the topic under study?
-Is public health relevance addressed?

Reviewer #1: not quite

Editorial and Data Presentation Modifications?

Use this section for editorial suggestions as well as relatively minor modifications of existing data that would enhance clarity. If the only modifications needed are minor and/or editorial, you may wish to recommend "Minor Revision" or "Accept".

Reviewer #1: no

Summary and General Comments

Reviewer #1: Authors have mostly answered my comments in a satisfactory manner but the issue remains on the scaling of F by linear body weight mg/kg. If the model is to be extrapolated across wider size ranges than those studied here, it is physiologically more plausible to scale by BSA (e.g. see https://pubmed.ncbi.nlm.nih.gov/33560094/) because gastric surface area scales with BSA. Whilst authors are unlikely to see a radically different fit with this model in their volunteer data, if the model is to be used to extrapolate e.g. to children, it is important covariates are mechanistically more plausible.

Thank your for this additional comment.

There seems to be a misunderstanding. We state clearly on lines 162 and 219 that elimination clearance (CL) scales allometrically with body weight, which is indeed a non-linear relationship. This is the most common way to account for the non-linear relationship between body weight and drug exposure and would allow for our results to be extrapolated to a paediatric population. Furthermore, this function resembles a BSA function very closely, but with the advantage that patient height is not needed (a variable that sometimes are not available in rural settings where this treatment is to be implemented). See difference below between body weight scaled based on allometry vs BSA.

Additionally, dose (as mg/kg) was implemented as an exponential covariate on CL and relative bioavailability (F) – also these are non-linear relationships. We have already defined these relationships in equation 1 and 2 in the manuscript (see below). We believe that these non-linear relationships define the biology of saturated absorption and enzyme-related elimination, and would be applied to all patients, irrespectively of age. Hopefully these clarifications are sufficient to address the concerns raised by the reviewer.

$$F_i = F \times e^{\theta_{Dose\_F} \times (Dose_i - Dose_{median})} \quad \text{(Eq.1)}$$

$$CL_i/F_i = CL/F \times e^{\theta_{Dose\_CL} \times (Dose_i - Dose_{median})} \quad \text{(Eq.2)}$$

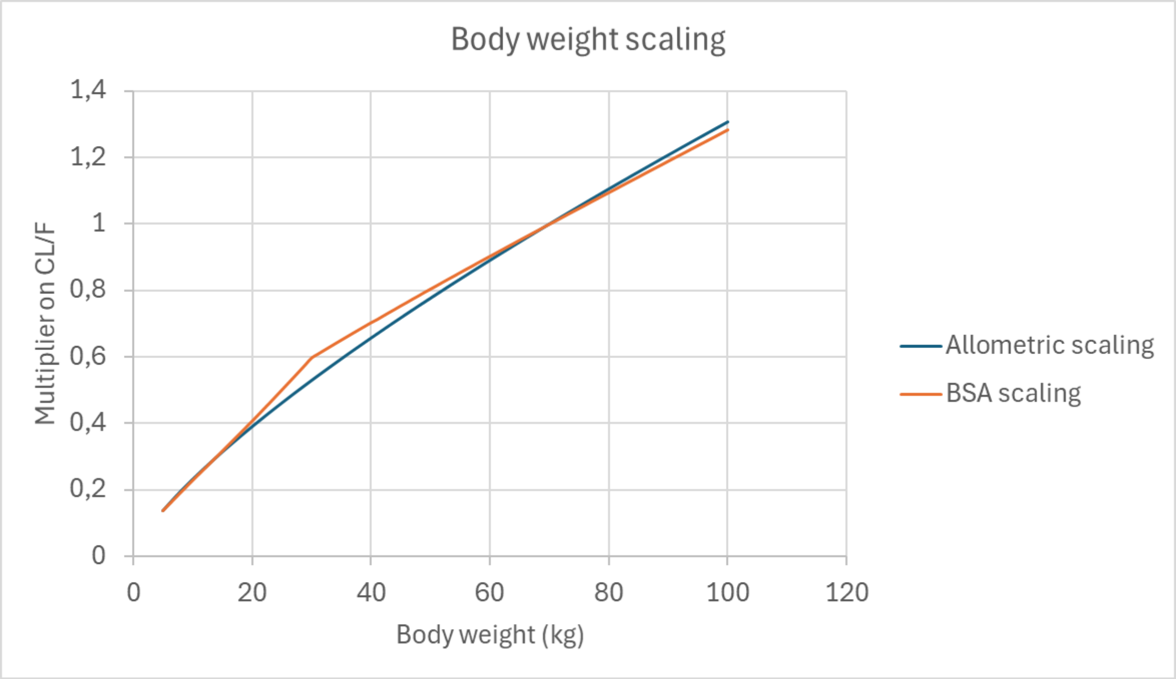

Supplement: Attachment — Submitted filename: Response_to_Reviewers2.pdf [file pntd.0014220.s006.pdf]
